# B3 Transcription Factors Determine Iron Distribution and *FERRITIN* Gene Expression in Embryo but Do Not Control Total Seed Iron Content

**DOI:** 10.3389/fpls.2022.870078

**Published:** 2022-05-06

**Authors:** Susana Grant-Grant, Macarena Schaffhauser, Pablo Baeza-Gonzalez, Fei Gao, Geneviève Conéjéro, Elena A. Vidal, Frederic Gaymard, Christian Dubos, Catherine Curie, Hannetz Roschzttardtz

**Affiliations:** ^1^Facultad de Ciencias Biológicas, Pontificia Universidad Católica de Chile, Santiago, Chile; ^2^IPSiM, Univ. Montpellier, CNRS, INRAE, Institut Agro, Montpellier, France; ^3^Centro de Genómica y Bioinformática, Facultad de Ciencias, Ingeniería y Tecnología, Universidad Mayor, Santiago, Chile; ^4^Escuela de Biotecnología, Facultad de Ciencias, Ingeniería y Tecnología, Universidad Mayor, Santiago, Chile; ^5^Agencia Nacional de Investigación y Desarrollo ANID-Millennium Science Initiative Program, Millennium Institute for Integrative Biology (iBio), Santiago, Chile

**Keywords:** B3 transcription factors, iron, seed, Arabidopsis, ferritin

## Abstract

Iron is an essential micronutrient for humans and other organisms. Its deficiency is one of the leading causes of anemia worldwide. The world health organization has proposed that an alternative to increasing iron content in food is through crop biofortification. One of the most consumed part of crops is the seed, however, little is known about how iron accumulation in seed occurs and how it is regulated. B3 transcription factors play a critical role in the accumulation of storage compounds such as proteins and lipids. Their role in seed maturation has been well characterized. However, their relevance in accumulation and distribution of micronutrients like iron remains unknown. In *Arabidopsis thaliana* and other plant models, three master regulators belonging to the B3 transcription factors family have been identified: FUSCA3 (FUS3), LEAFY COTYLEDON2 (LEC2), and ABSCISIC ACID INSENSITIVE 3 (ABI3). In this work, we studied how seed iron homeostasis is affected in B3 transcription factors mutants using histological and molecular approaches. We determined that iron distribution is modified in *abi3*, *lec2*, and *fus3* embryo mutants. For *abi3-6* and *fus3-3* mutant embryos, iron was less accumulated in vacuoles of cells surrounding provasculature compared with wild type embryos. *lec2-1* embryos showed no difference in the pattern of iron distribution in hypocotyl, but a dramatic decrease of iron was observed in cotyledons. Interestingly, for the three mutant genotypes, total iron content in dry mutant seeds showed no difference compared to wild type. At the molecular level, we showed that genes encoding the iron storage ferritins proteins are misregulated in mutant seeds. Altogether our results support a role of the B3 transcription factors ABI3, LEC2, and FUS3 in maintaining iron homeostasis in Arabidopsis embryos.

## Introduction

Iron plays an essential role in cells, due to its role as a cofactor in many proteins. Although iron is one of the most abundant elements in the earth’s crust, it is the least bioavailable, especially in calcareous or alkaline soils (Marschner, 1995). In plants, iron is mostly required in mitochondria and chloroplasts, where it is mainly used in the electron transport chain of respiration and photosynthesis, respectively (Jeong and Guerinot, 2009). Iron deficiency in plants causes reduction of vegetative growth and yield loss in crops (Abadía et al., 2011). In humans, iron deficiency is one of the leading causes of anemia worldwide, affecting about one billion people, mainly pregnant women and preschool children of underdeveloped countries (World Health Organization).^1^ One of the world health organization objectives of global nutrition for 2025 is to reduce anemia in women of childbearing age by 50%. In pursuance of this aim, it is proposed to increase the iron intake through the biofortification of crops and food (Murgia et al., 2012).

Seeds are a significant iron sink in plants and they are one of the primary source of iron for animal nutrition. During plant evolution and also by anthropogenic causes, iron accumulation and iron distribution in seeds has been modified (DeFries et al., 2015; Ibeas et al., 2019; Vidal Elgueta et al., 2021). For humans, grains contribute half of the dietary iron (Guerinot and Yi, 1994; Trumbo et al., 2001). During maturation stages of seed development, embryos are loaded with iron (Ravet et al., 2009; Roschzttardtz et al., 2009). Little information is available about the mechanisms involved in iron acquisition and accumulation by embryonic cells. Grillet et al. (2014) reported that ascorbate efflux from *Arabidopsis thaliana* embryos could be necessary for the reduction of Fe^3+^ previous to its uptake by the embryo. Citrate, a weak organic acid known to be one of the main iron ligands, has been suggested to play a role in iron uptake by germinating *Arabidopsis thaliana* embryos (Roschzttardtz et al., 2011).

In seeds, iron is stored in vacuoles, unlike vegetative tissues where the main pool of iron is found in other subcellular compartments including plastids (leaf mesophyll, pollen grains) and parietal space (root vascular cylinder) (Roschzttardtz et al., 2009, 2013). Two methods have been widely used to visualize iron in the seed: synchrotron radiation micro X-ray fluorescence (SμXRF), and a histochemical method based on the Perls/DAB dye (Roschzttardtz et al., 2009). These methods have shown that iron accumulates in the vacuoles of the endodermis cell layer during seed maturation (Kim et al., 2006; Roschzttardtz et al., 2009; Mary et al., 2015).

One of the major transporters allowing the entrance of iron into the endodermal vacuoles is the tonoplast-localized VACUOLAR IRON TRANSPORTER 1 (VIT1) (Kim et al., 2006). So far, VIT1 is the only player described to be involved in iron acquisition and distribution during the maturation of Arabidopsis embryos. *VIT1* transcripts are transiently accumulated during embryo maturation and decrease at the mature stage (Kim et al., 2006). In *vit1* mutant embryos, iron is mislocalized into the subepidermal cells of the cotyledons (Kim et al., 2006; Roschzttardtz et al., 2009; Chu et al., 2017; Eroglu et al., 2017; Höller et al., 2022). Also, *vit1* grows poorly compared to wild type seeds in alkaline soil where iron is scarcely available, indicating that proper iron distribution in the embryo is crucial for iron metabolism during germination (Kim et al., 2006; Roschzttardtz et al., 2009; Mary et al., 2015). During seed germination, transcripts encoding NATURAL RESISTANCE ASSOCIATED MACROPHAGE PROTEIN 3 and 4 (NRAMP3 and NRAMP4) transporters accumulate. NRAMP3 and NRAMP4 are responsible for iron remobilization from the vacuoles of endodermis cells during post-germinative growth (Lanquar et al., 2005). Consequently, *nramp3 nramp4* double mutant plants cannot remobilize the iron accumulated in the embryo, thereby slowing down post-germinative development (Lanquar et al., 2005).

Ferritins are important players in iron homeostasis. Ferritins form multimeric complexes composed of 24 subunits organized in a sphere shape, and each complex can store up to 4.500 atoms of iron in its central cavity (Guerinot and Yi, 1994; Ravet et al., 2009). By complexing iron, ferritins play an essential role in reducing oxidative damage in the cell. Importantly, iron excess increases ferritin abundance in roots and shoots (Reyt et al., 2015). Therefore, the expression of *FERRITIN* genes is used as a proxy for iron availability in the cell. No reports have been published about the genetic control of *FERRITIN* gene expression during seed development. In Arabidopsis, four genes encode FERRITIN proteins, and *AtFER2* is the only one expressed in the late maturation stages of seed development, during iron loading. AtFER2 protein has been detected in desiccated seeds, while AtFER1, AtFER3, and AtFER4 proteins are detected before the maturation stage of seed development and after germination. Interestingly, *Atfer2* mutant seeds have a low germination rate. Also, *Atfer2* seeds are more susceptible to pro-oxidant agents during seed germination and early post-germinative development (Ravet et al., 2009), confirming the role of AtFER2 in cell protection against oxidative stress.

Like proteins, sugars, and lipids, iron is stored in seed for later utilization by the germinating plantlet (Lanquar et al., 2005; Kim et al., 2006; Roschzttardtz et al., 2009). If the overall genetic regulation of iron uptake is quite well described and understood (Gao et al., 2019; Gao and Dubos, 2021), little information for seed iron loading and distribution is available (Roschzttardtz et al., 2020; Sun et al., 2021). Several transcription factors have been involved in the regulation of gene expression during seed development. Among them, B3 transcription factors, like FUSCA3 (FUS3), LEAFY COTYLEDON2 (LEC2), and ABSCISIC ACID INSENSITIVE3 (ABI3) play a pivotal role during embryo maturation (Ooms et al., 1993; Meinke et al., 1994; Kroj et al., 2003; To et al., 2006; Santos-Mendoza et al., 2008; Carbonero et al., 2017). Mutants for these transcription factors accumulate less seed storage compounds (sugars, lipids, and proteins). Mutants of B3 transcription factors show different phenotypes, such as: ectopic trichomes (*lec2*), accumulation of anthocyanins in cotyledons (*fus3* and *lec2*), the inability of degrading chlorophyll (*abi3* and *lec2*), and intolerance to desiccation (*abi3* and *fus3*) (To et al., 2006).

In this work, we sought to determine whether the FUS3, LEC2, and ABI3 transcription factors control the pattern of iron stores in seed. To that aim, we studied how seed iron homeostasis is affected in B3 transcription factors mutants using histological and molecular approaches. We determined that iron distribution is modified in *abi3*, *lec2*, and *fus3* embryo mutants. However, total iron content in dry mutant seeds showed no difference compared to wild type. This result suggests, at least in what was evaluated by the effect of the studied mutants, that the embryo does not control how much iron is delivered in seed during seed development. At the molecular level, we showed that genes encoding ferritins are misregulated in mutant seeds. Finally, we propose a genetic model involving B3 transcription factors ABI3, LEC2, and FUS3 in iron homeostasis in Arabidopsis embryos.

## Results

### Iron Distribution and Iron Total Content in *Lec2*, *Abi3*, and *Fus3* Seed Mutants

**FIGURE 1:**
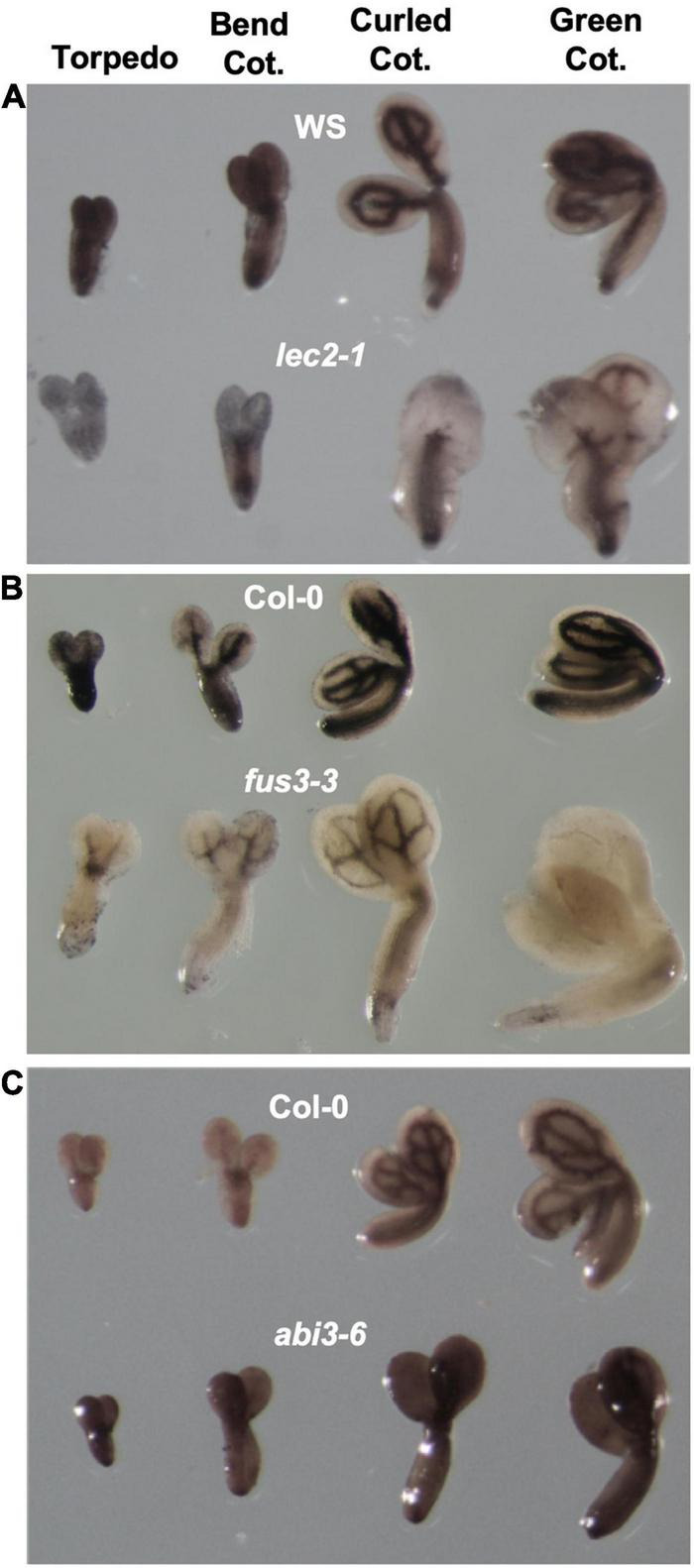
Iron distribution during embryo development in B3 transcription factors mutants. Perls/DAB staining was performed in isolated embryos from torpedo to green cotyledon stages. In each image the upper rows show wild-type embryos and the lower rows represent B3 transcription tractor mutants. **(A–C)** Show *lec2-1*, *fus3-3*, and *abi3-6*, respectively. Cot, cotyledon.

**FIGURE 2:**
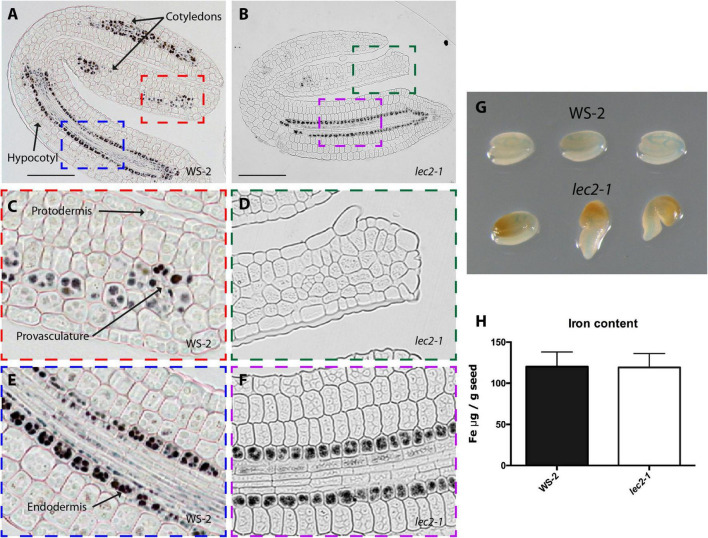
Iron distribution and accumulation in *lec2-1* mutants. Histological section of dry seed of *Arabidopsis thaliana* WS-2 **(A,C,E)** and *lec2-1* **(B,D,F)** stained with Perls/DAB. **(C,D)** Cotyledons and **(E,F)** hypocotyl zoomed zones highlighted in colors in A and B. Bars = 100 μm. **(G)** Perls staining in whole embryo, in the top row three WS-2 embryos, in the bottom row three *lec2-1*. **(H)** Iron content in dry seed of WS-2 and *lec2-1*. Four samples were analyzed for each genotype.

**FIGURE 3:**
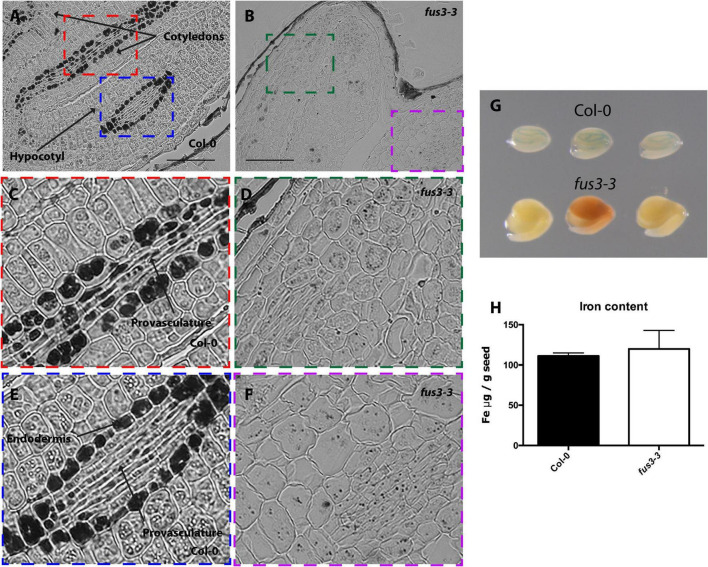
Iron distribution and accumulation in *fus3-3* mutants. Histological section of dry seed of *Arabidopsis thaliana* Col-0 **(A,C,E)** and *fus3-3* **(B,D,F)** stained with Perls/DAB. **(C,D)** Cotyledons and **(E,F)** hypocotyl zoomed zones highlighted in colors in A and B. Bars = 100 μm. **(G)** Perls staining in whole embryo, in the top row three Col-0 embryos, in the bottom row three *fus3-3*. **(H)** Iron content in dry seed of Col-0 and *fus3-3*. Four samples were analyzed for each genotype.

**FIGURE 4:**
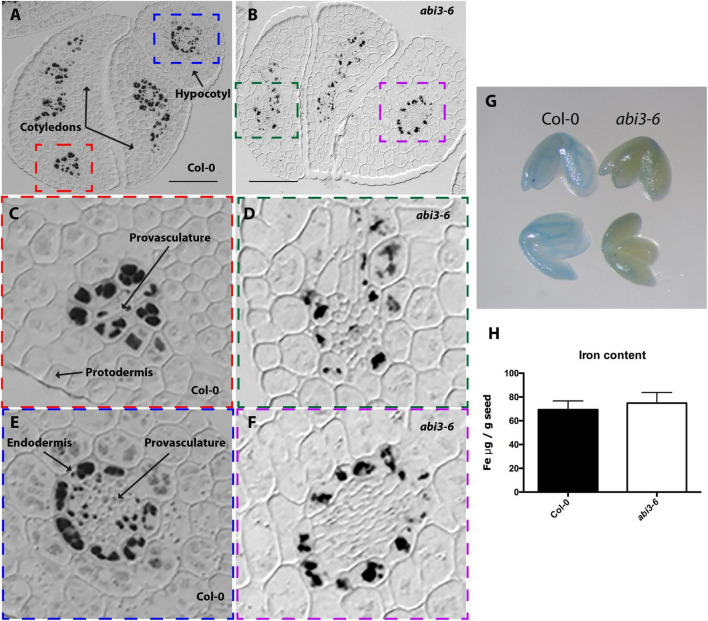
Iron distribution and accumulation in *abi3-6* mutants. Histological section of dry seed of *Arabidopsis thaliana* Col-0 **(A,C,E)** and *abi3-6* **(B,D,F)** stained with Perls/DAB. **(C,D)** Cotyledons and **(E,F)** hypocotyl zoomed zones highlighted in colors in A and B. Bars = 50 μm. **(G)** Perls staining in whole embryo, in the left Col-0 embryos, in the right *abi3-6* embryos. **(H)** Iron content in dry seed of Col-0 and *abi3-6*. Four samples were analyzed for each genotype.

We first determined if ABI3, LEC2, and FUS3, are involved in iron distribution in Arabidopsis seed by examining the territories and amount of iron pools in seeds of the corresponding mutants. For this, mutant embryos were isolated from four stages of embryo maturation: torpedo, bend, curled and mature green cotyledon (Figure 1) and stained for iron using the Perls/DAB method. No differences in iron distribution were observed in WT embryos from three different analyzed ecotypes, Col-0, WS and Ler, where iron is detected in cotyledons and hypocotyl following the provasculature patterning (Figure 1 and Supplementary Figure 1). In *lec2-1* embryos, the pattern and amount of iron staining was unchanged in the hypocotyl. In cotyledons however, the iron amount was markedly reduced in embryos of the *lec2-1* mutant compared to WT at the different maturation stages studied (Figures 1A, 2B,D,G). Iron was also less detected in *fus3-3* embryos at all seed maturation stages compared with WT, and in the mature green cotyledon stage iron was almost undetected (Figures 1B, 3B,D,F,G). Finally, in *abi3-6* mutant embryos, the pattern of iron was not affected but the intensity of the staining in the provasculature zone was greatly reduced compared to WT embryos. This observation suggests that iron is less accumulated in the *abi3-6* mutant embryos (Figures 1C, 4B,D,F,G). Different mutant alleles for *abi3* were included in the study, including the weak allele *abi3-1* and the strong allele *abi3-5*. *abi3-5*, like *abi3-6*, showed a loss of iron accumulation that follows provasculature compared with wild type embryos (Supplementary Figure 1A).

In order to determine where iron accumulates at the cell and subcellular level in dry mutant embryos, thin sections from isolated mutant embryos were stained by Perls/DAB. Wild type embryos accumulated iron in vacuoles of the endodermis and surrounding provasculature cells (Figures 2A, 3A, 4A and Supplementary Figure 1; Roschzttardtz et al., 2009). In *lec2-1*, iron was also detected in vacuoles of endodermal cells in the hypocotyl. However, no iron was detected in *lec2-1* cotyledons (Figures 2B,D). To verify that the decrease of Perls-DAB signal did correspond to an actual reduction of iron content, we used the more quantitative method of staining based on the Perls dye alone (see Roschzttardtz et al., 2009). Perls staining on isolated dry seed embryos showed a weaker blue signal in the provasculature of *lec2-1* cotyledons compared with wild type, confirming that *lec2-1* embryos accumulate less iron in this region (Figure 2G). However, we found no decrease of total iron content in *lec2-1* dry seeds (Figure 2H). Further analysis may be carried out in order to determine if the hypocotyl endodermal iron pool is larger in *lec2-1* mutants compared to the WT. In *fus3-3* mature embryo sections, iron was detected in several cell layers, including cortex, endodermis and provasculature cells (Figures 3C,D), and Perls/DAB staining was less intense compared with WT embryos stained sections. Interestingly, iron was detected in structures that appear to be distinct from vacuoles (Figures 3D,F). Like for the *lec2* mutant, Perls staining confirmed that *fus3-3* dry seed embryos contain less iron than WT embryos (Figure 3G). Like for *lec2-1*, total seed iron content was not affected by the mutation of *FUS3* (Figure 3H).

Finally, we analyzed the impact of ABI3 in iron distribution. To that aim we stained with perls/DAB sections of dry embryos from three *abi3* mutant alleles, *abi3-6* (Figure 4), *abi3-1* and *abi3-5* (Supplementary Figures 1, 4). In all three alleles, iron was less concentrated in the vacuoles of cells that surround provasculature both in hypocotyl and in cotyledons (Figures 4B,D,F and Supplementary Figure 1). In *abi3-5* sections, Perls/DAB staining suggests an increased number of iron-FERRITIN complexes in cortex cells, whereas almost no vacuolar iron in endodermis is detected (Supplementary Figure 1). Total iron content was determined for *abi3-6* seeds, and no difference in total iron amount was observed compared with wild type dry seeds (Figure 4H).

### FERRITIN-Encoding Genes Are Deregulated in B3 Transcription Factor Mutant Seeds

**FIGURE 5:**
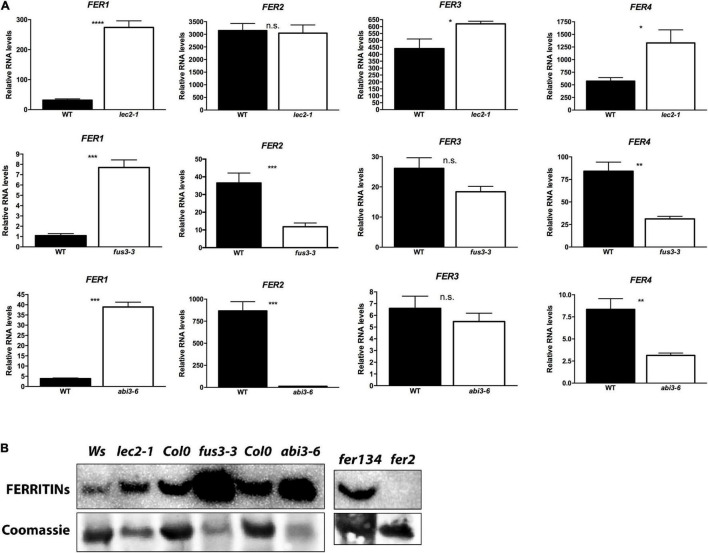
FERRITINs are deregulated in B3 transcription factors mutants. **(A)** *FERRITINs* transcripts accumulation in green cotyledon stage (M) and in dry seeds (D). *T*-test was performed, *P*-value < 0.05, *p < 0.05, **p < 0.01 and ***p < 0.001. **(B)** Protein accumulation of FERRITINs in dry seeds. 10 μg of proteins were loaded in each well. Poly-clonal antibody detecting all four FERRITINs was used. As loading control the membrane was stained with Coomassie Blue.

The analysis of *fus3-3* and *abi3-5* dry seed embryo sections stained by Perls/DAB revealed that iron accumulates in dots that are clearly distinct from vacuolar iron stores visible in the wild type embryos. Because iron-ferritin, another important store of iron in plant tissues, appears as dot-like structures, we hypothesized that the Perls/DAB stained dots in *fus3* and *abi3* are indeed iron-FERRITIN complexes (Figures 3D,F and Supplementary Figure 1). These complexes were detected previously in leaves from iron excess treated plants (Divol et al., 2013; Roschzttardtz et al., 2013). Ravet et al. (2009) studied the kinetics of accumulation of the 4 isoforms of ferritin of Arabidopsis during seed development. AtFER1, AtFER3, and AtFER4 accumulate during morphogenesis stages and are not detected during seed maturation. In contrast, AtFER2 is the only ferritin detected in mature seeds. In order to evaluate if *FERRITIN* encoding genes are misregulated in *lec2-1*, *fus3-3*, and *abi3-6* mutants, we performed qRT-PCR analysis using total RNA from dry seeds (Figure 5A). Overall, all four *AtFER* genes were misregulated in the B3 transcription factor mutants, except for *AtFER2* in *lec2-1* and *AtFER3 in fus3-3 and abi3-6* (Figure 5A). In order to corroborate our findings, we obtained transcriptomics datasets from seeds of WT and B3 mutants (Yamamoto et al., 2014, GSE61686) and determined gene expression of Ferritins. As shown in Supplementary Figure 2, similar results were found for Ferritins in *lec2-1*, *fus3-3*, and *abi3-6* in these samples. Interestingly, *AtFER1* transcript, encoding the main ferritin isoform of Arabidopsis strongly responsive to iron excess, was strongly accumulated in the seed of all three B3 transcription factor mutants analyzed (Figure 5A). To determine if the changes observed in the accumulation of *AtFER* transcripts are correlated with changes in AtFER polypeptides level, we performed a Western blot, using an antiserum against Arabidopsis ferritins. We included the *fer2* mutant, which serves as a negative control for the presence of FER2 in seeds, and the *fer134* mutant, which serves as positive control for AtFER2 presence (Figure 5B). For all the samples, we were able to detect AtFER polypeptides, except in *fer2*, confirming that FER2 is the only ferritin produced in WT mature seed. Total ferritin proteins were increased in *lec2-1, fus3-3*, and *abi3-6* mutant seeds (Figure 5B). It is likely caused by an increase of AtFER1 accumulation in the mutant seeds since (1) *AtFER2* transcripts level was either unchanged or decreased in the three mutants while in the meantime (2) the level of *AtFER1* transcripts was almost 10-fold increased.

## Discussion

Using Arabidopsis and other dicot model plants like *Brassica napus* and *Chenopodium quinoa*, it has been shown that iron accumulates in embryo during seed maturation (Roschzttardtz et al., 2009; Ibeas et al., 2017, 2019). However, how this process is regulated genetically remains elusive. As a gene candidate approach and using *Arabidopsis thaliana* as a plant model, we focused our study on mutants of the B3 transcription factors family like LEC2, FUS3, and ABI3, known as being master regulators of embryo maturation gene expression and also regulators of the setting up of the seed reserves (Santos-Mendoza et al., 2008). Using Perls/DAB staining we were able to determine the pattern of iron accumulation in mutant embryos (Figures 1–4). Iron distribution in the different embryo mutants showed dramatic differences compared with that of WT embryos, indicating that LEC2, FUS3, and ABI3 transcription factors play a crucial role in iron distribution in embryos. Interestingly, total iron content in mutant seeds was not affected (Figures 1H, 2H, 3H)*.* Additional and more quantitative analyses should be carried out in order to determine where iron accumulates in the mutant seeds in non-embryonic tissues. The fact that total iron was not modified in the mutant seeds suggests that iron could be accumulated in others seed structures like integuments. In the case of *lec2-1*, our experimental approach does not allow determining if the hypocotyl endodermal iron pool is larger in the mutant compared to the WT. Unlike other non-mineral nutrients that are synthesized and accumulated in the seeds, iron is transported into the seed from the maternal tissue, suggesting that the maternal tissue determines the amount of iron delivered to the seed independently of where iron accumulates in the embryo. Using synchrotron X-ray fluorescence microtomography, Kim et al. (2006) showed that in the embryo of the *vit1* mutant, iron is mislocalized. Interestingly, total iron content in *vit1* seeds is unchanged, as in mutants of the B3 transcription factors. All these results strongly indicate that ABI3, LEC2, and FUS3 transcription factors regulate iron distribution in the embryo whereas they do not control iron loading from maternal tissues. Nevertheless, how B3 transcription factors modulate iron distribution in embryos is still to be determined. Whether this is by directly modulating the expression of iron transporters is an opened question. Conversely, defects in iron uptake, as is the case for the *bhlh121* loss-of-function mutants (for the *bHLH121* master transcriptional regulator of iron homeostasis and *AtFER1*, *AtFER3*, and *AtFER4* expression), leads to decrease in seed iron content without affecting iron distribution (Gao et al., 2020a,b). Recently, it has been described in Arabidopsis that INO, a member of the YABBY transcription factors family, regulates negatively seed iron loading. A reduced expression of INO increased iron accumulation in the embryo without changing its distribution pattern (Sun et al., 2021).

**FIGURE 6:**
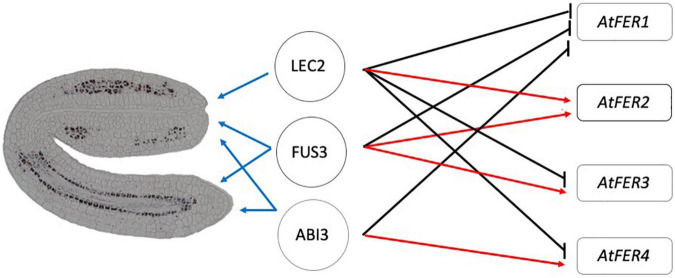
Model involving LEC2, FUS3, and ABI3 transcription factors in iron embryo loading and FERRITINs gene expression. *AtFER1* gene expression is negatively regulated by LEC2, FUS3, and ABI3. *AtFER2* gene expression is positively regulated by LEC2 and FUS3. *AtFER3* and *AtFER4* are positively regulated by FUS3 and ABI3, and negatively regulated by LEC2. LEC2 is involved in iron loading in cotyledons, whilst FUS3 and ABI3 are involved in iron loading in whole embryo. Black arrows, negative regulation; Red arrows, positive regulation; Blue arrows, where transcription factor acts in the embryo.

Perls/DAB staining allowed us to observe where iron is localized in the mutant embryos at the subcellular level. Iron was detected in dots resembling iron-ferritin complexes (Divol et al., 2013; Roschzttardtz et al., 2013; Figures 3C,D and Supplementary Figure 1). Iron-ferritin complexes have been detected in seed from several plant species, but in Arabidopsis no more than 5% of total iron in seed is found associated to ferritin (Briat et al., 2010; Moore et al., 2018). Ferritin-less mature seeds, obtained by mutating the *AtFER2* gene, have identical iron content than WT seeds (Ravet et al., 2009; Briat et al., 2010). We confirmed by different approaches that ferritins are misregulated in *lec2-1, fus3-3*, and *abi3-6* seeds at the transcript and protein levels (Figure 5). The qRT-PCR analysis suggested that AtFER1 could be the major ferritin form accumulated in the *lec2-1, fus3-3*, and *abi3-6* mutants seeds, but we could not confirm this at the protein level because anti-ferritin immunodetection does not allow discriminating between the four types of ferritin polypeptides in Arabidopsis. It will be interesting to determine spatial expression pattern of specific ferritins and other relevant genes involved in iron transport, on the embryo tissues and its correlation with the iron accumulation. Ferritins are deregulated at the transcript and the protein levels in *lec2* mutant seeds (Figure 5); however, iron-ferritin complexes were not detected in the *lec2* mutant embryo by Perls/DAB staining (Figure 2). We could speculate that: (i) iron is not available to form complexes with ferritins in *lec2* embryos, implying that vacuoles of endodermis cells in hypocotyl, expressing *VIT1* transporter, are efficiently depleting iron from all cell embryos, hence preventing iron-ferritin complex formation; or (ii) that ferritins are accumulating in other seed structures like integuments. In both cases, it will be interesting to perform a ferritin immunodetection on embryo sections in order to decipher where ferritins are accumulated in *lec2* mutant and WT seeds. From a genetic point of view, our results indicate that LEC2, FUS3, and ABI3 are negative regulators of *AtFER1*, FUS3, and LEC2 are positive regulators of *AtFER2*, LEC2 is a negative regulator of *AtFER3*, and lastly, *AtFER4* is negatively regulated by LEC2 and positively regulated by FUS3 and ABI3 (Figures 5, 6). Unlike FUS3 and ABI3, that seem to regulate iron accumulation in the whole embryo, LEC2 is controlling iron accumulation in cotyledons (Figures 1–4, 6). In order to determine whether B3 transcription factors directly control the expression of *FERRITIN* genes, we generated a gene regulatory network (GRN) composed of genes whose expression is differentially expressed in *abi3*, *fus3*, or *lec2* mutants (Supplementary Table 2). We searched for possible transcription factor-target interaction data between differentially expressed genes in the CisBP database (Weirauch et al., 2014), the Plant Cistrome Database (O’Malley et al., 2016) and in the Arabidopsis Gene Regulatory Interaction Server (Yilmaz et al., 2011). To narrow our GRN to genes that may be interacting in the context of seed development, we calculated coexpression between transcription factor-target pairs based on transcriptomics data from developing seeds obtained from AtGenExpress (Schmid et al., 2005). The resulting GRN contains 267 nodes and 762 edges (Supplementary Table 3). We found evidence for direct interaction of B3 transcription factors for 158 genes, of which 86 have an associated GO term related to iron according to the Thalemine database (Supplementary Table 4). Concerning B3 regulation of *FERRITIN* genes, we found evidence for direct control of *FER2* by ABI3 and LEC2 and of *FER4* by ABI3 and FUS3 (Supplementary Figure 3). In accordance with these findings, using the Eukaryotic Promoter Database (Dreos et al., 2017),^2^ we found ABI3 and LEC2 binding motifs in the *FER2* promoter (at −1668, −1667, −1549, and −557 for ABI3 and −1822, −1821, −1666, −535 for LEC2, considering −2000 to 100 bp from the *FER2* transcriptional start site) and ABI3 and FUS3 binding motifs in the *FER4* promoter (at −1138, −1139, −1219, −1220, −1741, −1750 for ABI3 and −1138, −1139, −1219, −1220, −1587, −1588, −1750, −1751 for FUS3, considering −2000 to 100 bp from the *FER4* transcriptional start site). In the case of *FER1* and *FER3*, no evidence for potential direct binding of B3 transcription factors was found; however, we were able to find an indirect potential regulation of *FER1* and *FER3* via the ethylene-related transcription factors EIL1, EIL3, and EIN3 that act downstream LEC2 and FUS3 (Supplementary Figure 3). Besides *FER1* and *FER3*, these ethylene-related transcription factors also target *FER2* and *FER4* either directly or by indirect control by transcription factors WRKY9 and TCP24 (Supplementary Figure 3). Finally, transcriptomic public data shows that other iron-related genes such as *VIT1* and *MTP8* iron transporters are misregulated in some of the B3 transcription factor seed mutants analyzed in this article (Supplementary Figure 4). For instance, *VIT1* is downregulated in *fus3-3* mutant, which could help explain the loss of iron accumulation in the vacuoles of the endodermis in this mutant. Further studies should be performed in order to describe how *FERRITIN* genes are regulated during seed development, and the mechanism by which LEC2, FUS3, and ABI3 regulate their expression.

## Materials and Methods

### Arabidopsis Growth Conditions

In this study, we used WT genotype Columbia-0 (Col-0) for *fus3-3* (CS8014) and *abi3-6* (SALK_138922), Wassilewskija (Ws) to *lec2-1* (CS2728), Landsberg (Ler) to *abi3-1* (CS24), and *abi3-5* (CS6131). All genotypes used in this article were obtained from ABRC or kindly provided by Dr. Xavier Jordana. All these genotypes were grown on soil and grown in a growth chamber (21^°^C, 16 h light/8 h dark). For the *fus3-3* and *abi3-6* mutants, were sown seeds at the green cotyledon stage directly on soil in order to obtain adult plants. The plants were irrigated with water as needed. Each mutant plant was grown at the same time and same conditions with its wild type ecotype.

### Embryo Fixation, Embedding, and Sectioning

To perform Perls/DAB staining, embryos were vacuum infiltrated with a solution with 4% w/v of paraformaldehyde in Na-phosphate buffer pH 7 for 1 h. Then, embryos were incubated overnight at room temperature. Fixated embryos were dehydrated with a serial bath of ethanol, 50, 60, 70, 80, 90, 95, and 100% v/v for 1 h per bath. Dehydrated embryos were incubated with ethanol/butanol overnight and then incubated again in butanol 100% overnight. A final incubation with butanol/resin overnight at room temperature was performed.

Then, embryos were embedded in Technovit 7100 resin (Kulzer) according to the manufacturer’s recommendation. Sections of 2–3 μm were obtained using a microtome.

### Perls Staining and DAB/H_2_O_2_ Intensification

Perls stain and DAB/H_2_O_2_ intensification were performed as described by Roschzttardtz et al. (2009). Briefly, embryos were dissected from silique or seed depending on whether we were analyzing maturation or seed. Seeds were imbibed in distilled water before dissecting. The embryos were vacuum infiltrated at room temperature for 45 min with Perls stain solution. Embryos were maintained in Perls solution until the image was taken.

For intensification, after Perls staining embryos were incubated for 45 min with a methanol solution that contained 0.01 w/v NaN_3_ and 0.03% v/v H_2_O_2_. Finally, after a wash with phosphate buffer 0.1 M pH 7.4, intensification was performed. Embryos were incubated with an intensification solution that contained phosphate buffer 0.1 M pH 7.4, 0.005 w/v DAB, 0.005 v/v H_2_O_2_ and 0.005 w/v CoCl_2_ for 5–10 min. Embryos were washed and maintained in distilled water to stop the reaction. Images were acquired using SMZ800 zoom stereomicroscope (Nikon). Sections, from at least 3 independent sets of stained embryos, were observed with a microscope Eclipse 80i (Nikon). In both cases, images were acquired with the camera Nikon Digital Sight DS-5M.

### Iron Quantification

For each genotype, 20–22 mg of dry seeds were digested with 3 mL of HNO_3_ 0.5 M and 1 mL H_2_O_2_. Then, samples were taken to 10 mL with HNO_3_ 0.5 M and analyzed in ICP-MS. Each replicate corresponds to seeds from individual plants.

### RNA Isolation and cDNA Synthesis

Total RNA was obtained from 20 to 25 mg of dry seeds. Dry seeds were frozen with liquid nitrogen and maintained in –80^°^C until needed. The tissue was homogenized with the FastPrep-24 instrument (MP Biomedicals) following the manufacturer recommendation.

The powder was used to isolate RNA using Spectrum Plant Total RNA kit (Sigma) according to manufacturer’s instructions.

After obtaining the RNA, 1 μg was used to digest the DNA residual with RQ1 RNase-Free DNase (Promega) according to manufacturer’s instruction. The synthesis of cDNA was then performed with the First Strand cDNA Synthesis kit (Thermo) following manufacturer recommendation.

### qPCR Analysis

Primers were designed using AmplifiX 1.6.3. Primers were designed to have a Tm of 60^°^C and to obtain amplicons between 100 and 150 bp long. Then, primers were used to make a standard curve (Supplementary Table 1). For qPCR procedures and standard curve, we used the StepOnePlus Real-Time PCR System (Thermo), and Brilliant III Ultra-Fast SYBR Green QPCR Master Mix (Agilent) according to manufacturer’s instruction. For qPCR reaction, we used 1/10 dilution of cDNA. As a housekeeping gene TIP41-like (AT4G34270) was used (Dekkers et al., 2012).

### Western Blot

Total proteins of dry seeds were extracted in the following manner. First, dry seeds (20 mg) were frozen with liquid nitrogen and homogenized using the FastPrep-24 instrument (MP Biomedicals) following the manufacturer recommendation. Then homogenized seeds were resuspended in 500 μL of urea/thiourea Buffer [7 M Urea, 2 M thiourea, 4% CHAMPS, 1% DTT in 30 μM Tris–HCl solution (pH 8.5)], and vortexed for 30 min at 4^°^C, centrifuged and the supernatant recover. Total proteins were measured using the Bradford method. All the following procedures were performed as Tissot et al. (2019). Immunodetection was performed using SUPERSIGNAL WEST PICO PLUS (Thermo Scientific).

### Transcriptomics Data Analysis in Seeds of B3 Mutants

Microarray data (ATH1) was downloaded from the Gene Expression Omnibus (GEO) database^3^ [accessions GSE61686 for data from seeds of B3 mutants and their corresponding WT controls (Yamamoto et al., 2014), and GSE5634 for developmental time series of siliques and seeds (Schmid et al., 2005)]. Data was processed with the R package affy (Gautier et al., 2004) v.1.72.0 and normalized using the Robust Multi Array (RMA) method (Irizarry et al., 2003). For B3 mutant data, the normalized data was used to perform two-way ANOVA analyses with a false discovery rate of 5%. For the ANOVA, we used a model considering the expression of a given gene Y as Yi = β0 + β1T + β2G + β3TG + ε, where β0 is the global mean; β1, β2, and β3 are the effects of Time (T), Genotype (G), and the TG interaction, and the variable ε is the unexplained variance. For the seed developmental time series, normalized data was used to determine coexpression between all gene pairs using the corr.test function from the R package psych (Revelle, 2021) v.2.1.9 (method = “pearson,” adjust = “fdr,” alpha = “0.05”).

### Gene Regulatory Network Construction

The list of differentially expressed genes (G and TG interaction according to the 2-way ANOVA analysis) was used to generate a Gene Regulatory Network. We gathered regulatory interactions between differentially expressed transcription factors and target genes from three different sources: the Plant Cistrome Database (O’Malley et al., 2016),^4^ the CIS-BP database (Weirauch et al., 2014)^5^ and the Arabidopsis Gene Regulatory Information Server AGRIS (Yilmaz et al., 2011).^6^ The regulatory interactions were further filtered by considering gene coexpression during seed development, considering a Pearson correlation of *r* ≤ −0.8 or *r* ≥ 0.8 and a corrected *p*-value of ≤ 0.01 (data from Schmid et al., 2005). The network was visualized in Cytoscape (Shannon et al., 2003).

## Data Availability Statement

The datasets presented in this study can be found in online repositories. The names of the repository/repositories and accession number(s) can be found in the article/Supplementary Material.

## Author Contributions

SG-G and HR conceived, designed the experiments, and wrote the manuscript. SG-G, MS, PB-G, FeG, GC, EV, and HR performed the experiments. SG-G, EV, FrG, CD, CC, and HR analyzed the data. CD, CC, and HR contributed to reagents and materials. All authors contributed to the article and approved the submitted version.

## Conflict of Interest

The authors declare that the research was conducted in the absence of any commercial or financial relationships that could be construed as a potential conflict of interest.

## Publisher’s Note

All claims expressed in this article are solely those of the authors and do not necessarily represent those of their affiliated organizations, or those of the publisher, the editors and the reviewers. Any product that may be evaluated in this article, or claim that may be made by its manufacturer, is not guaranteed or endorsed by the publisher.

## Footnotes

1
http://www.who.int/nutrition/topics/ida/en/

2
https://epd.epfl.ch//index.php

3
https://www.ncbi.nlm.nih.gov/geo/

4
http://neomorph.salk.edu/dap_web/pages/index.php

5
http://cisbp.ccbr.utoronto.ca/

6
https://agris-knowledgebase.org/

## Supplementary Material

Supplementary Figure 1 Iron detection in embryos of different *abi3* mutant alleles. **(A)** Perls/DAB staining in whole embryos of wild type (Ler), *abi3-1*, and *abi3-5*. **(B–F)** Perls/DAB staining on histological sections of dry seed embryos. **(B)** wild type (Ler). **(C,D)** *abi3-1* cotyledons and hypocotyls, respectively. **(E,F)** *abi3-5* cotyledons and hypocotyl, respectively. Bar = 50 μm.

Supplementary Figure 2 Expression of *FERRITIN* genes in B3 mutants. Transcriptome datasets from seeds of B3 mutants (Yamamoto et al., 2014) were obtained from the GEO database (GEO accession GSE61686). We show the log_2_ normalized expression for *FER1*, *FER2*, *FER3* and *FER4* in developing seeds dissected from siliques from the *abi3-6*, *fus3-3*, and *lec2-1* mutants. DAF: days after flowering. The asterisks show means that significantly differ between mutants and their respective WT control (*t*-test, *p* ≤ 0.05).

Supplementary Figure 3 Gene regulatory network controlling *FERRITIN* gene expression in seeds. We obtained a list of differentially expressed genes from seed transcriptome datasets of B3 mutants and searched for transcription factor-target gene interaction data in the plant cistrome database (O’Malley et al., 2016), the CIS-BP database (Weirauch et al., 2014) and the Arabidopsis Gene Regulatory Information Server AGRIS (Yilmaz et al., 2011) in order to build a regulatory network. Transcription factor-target gene pairs were further filtered by coexpression (expression correlation) during seed development (Schmid et al., 2005). From this network, we extracted regulatory interactions between *FERRITIN* genes and transcription factors and visualized the network in Cytoscape (Shannon et al., 2003). In the network, triangles represent transcription factors and rectangles represent genes. The edges connecting the nodes represent regulatory interactions from AGRIS (red edges) and CIS-BP (blue edges). Arrow edges represent positive regulation (activation) and T edges represent negative regulation (repression) based on expression correlation.

Supplementary Figure 4 Expression of VIT1 and MTP8 genes in B3 mutants. Transcriptome datasets from seeds of B3 mutants (Yamamoto et al., 2014) were obtained from the GEO database (GEO accession GSE61686). We show the log_2_ normalized expression for *VIT1* and *MTP8* in developing seeds dissected from siliques from the *abi3-6*, *fus3-3*, and *lec2-1* mutants. DAF: days after flowering. The asterisks show means that significantly differ between mutants and their respective WT control (*t*-test, *p* ≤ 0.05).
