# Supplementary figures and images for: B3 Transcription Factors Determine Iron Distribution and FERRITIN Gene Expression in Embryo but Do Not Control Total Seed Iron Content
Source: Front Plant Sci. 2022 May 6;13:870078. doi: 10.3389/fpls.2022.870078 (PMC9120844; doi:10.3389/fpls.2022.870078)

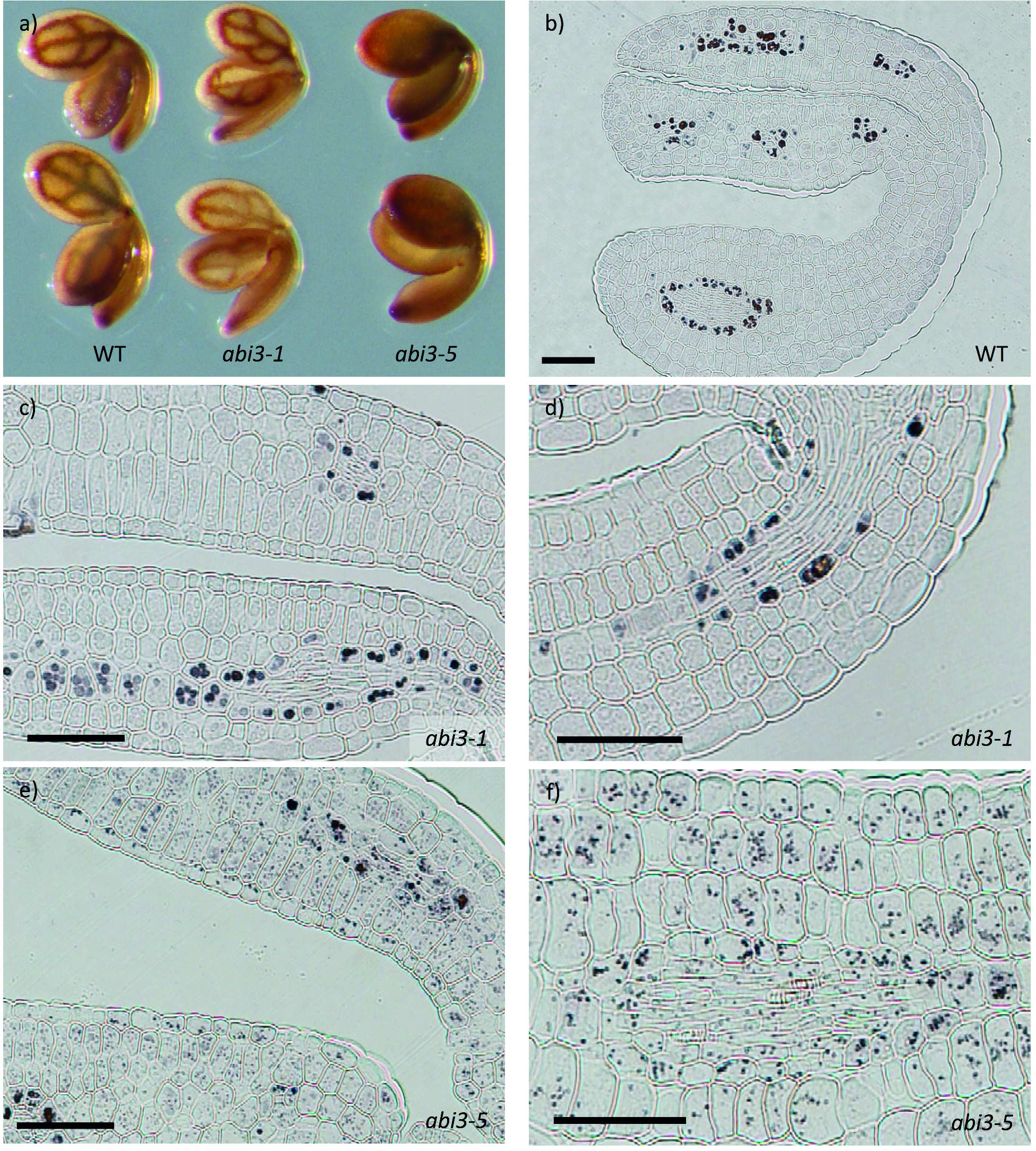

Supplement: Supplementary Figure 1 — Iron detection in embryos of different abi3 mutant alleles. (A) Perls/DAB staining in whole embryos of wild type (Ler), abi3-1, and abi3-5. (B–F) Perls/DAB staining on histological sections of dry seed embryos. (B) wild type (Ler). (C,D) abi3-1 cotyledons and hypocotyls, respectively. (E,F) abi3-5 cotyledons and hypocotyl, respectively. Bar = 50 μm. [file Image_1.JPEG]

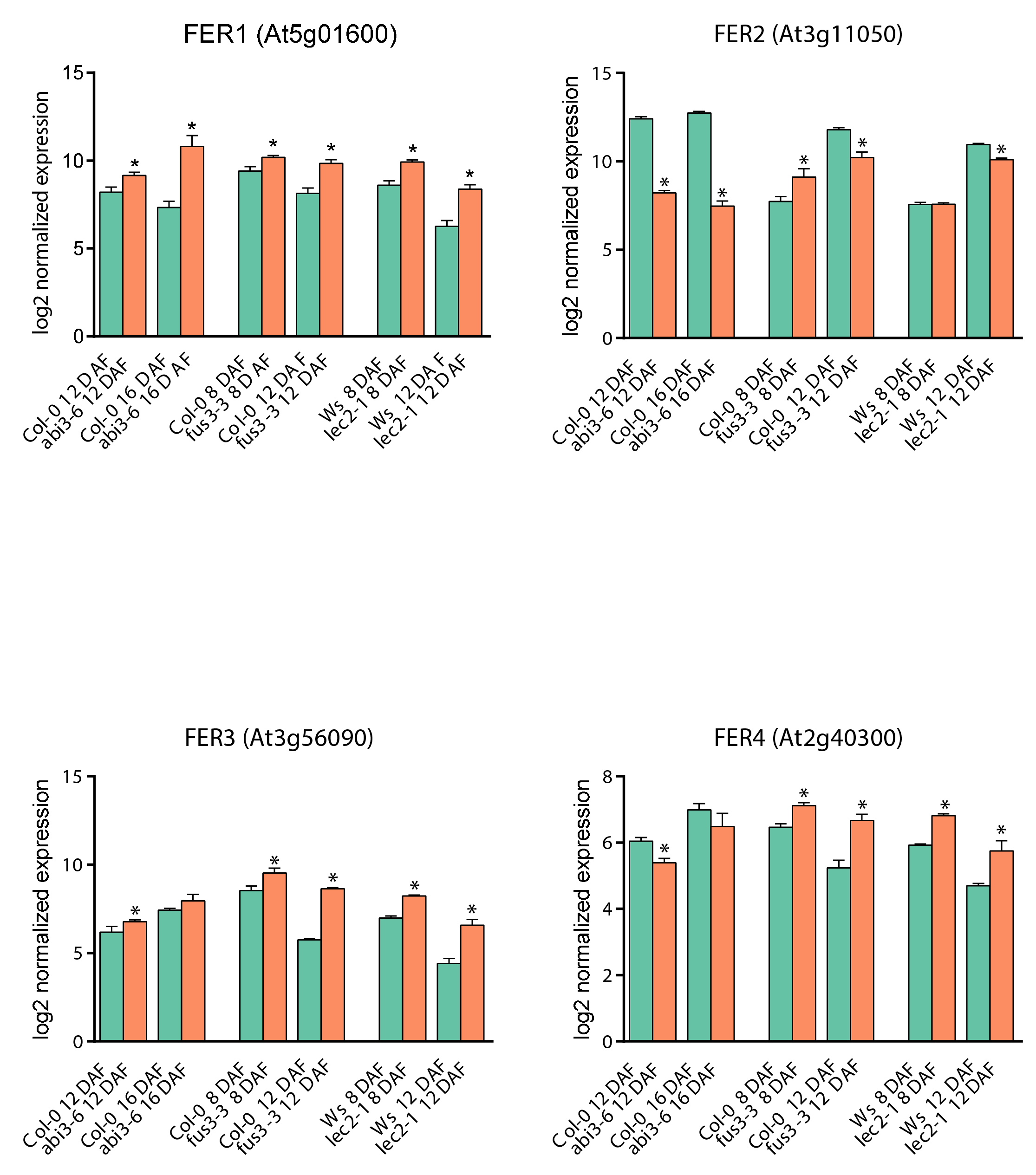

Supplement: Supplementary Figure 2 — Expression of FERRITIN genes in B3 mutants. Transcriptome datasets from seeds of B3 mutants (Yamamoto et al., 2014) were obtained from the GEO database (GEO accession GSE61686). We show the log2 normalized expression for FER1, FER2, FER3 and FER4 in developing seeds dissected from siliques from the abi3-6, fus3-3, and lec2-1 mutants. DAF: days after flowering. The asterisks show means that significantly differ between mutants and their respective WT control (t-test, p ≤ 0.05). [file Image_2.JPEG]

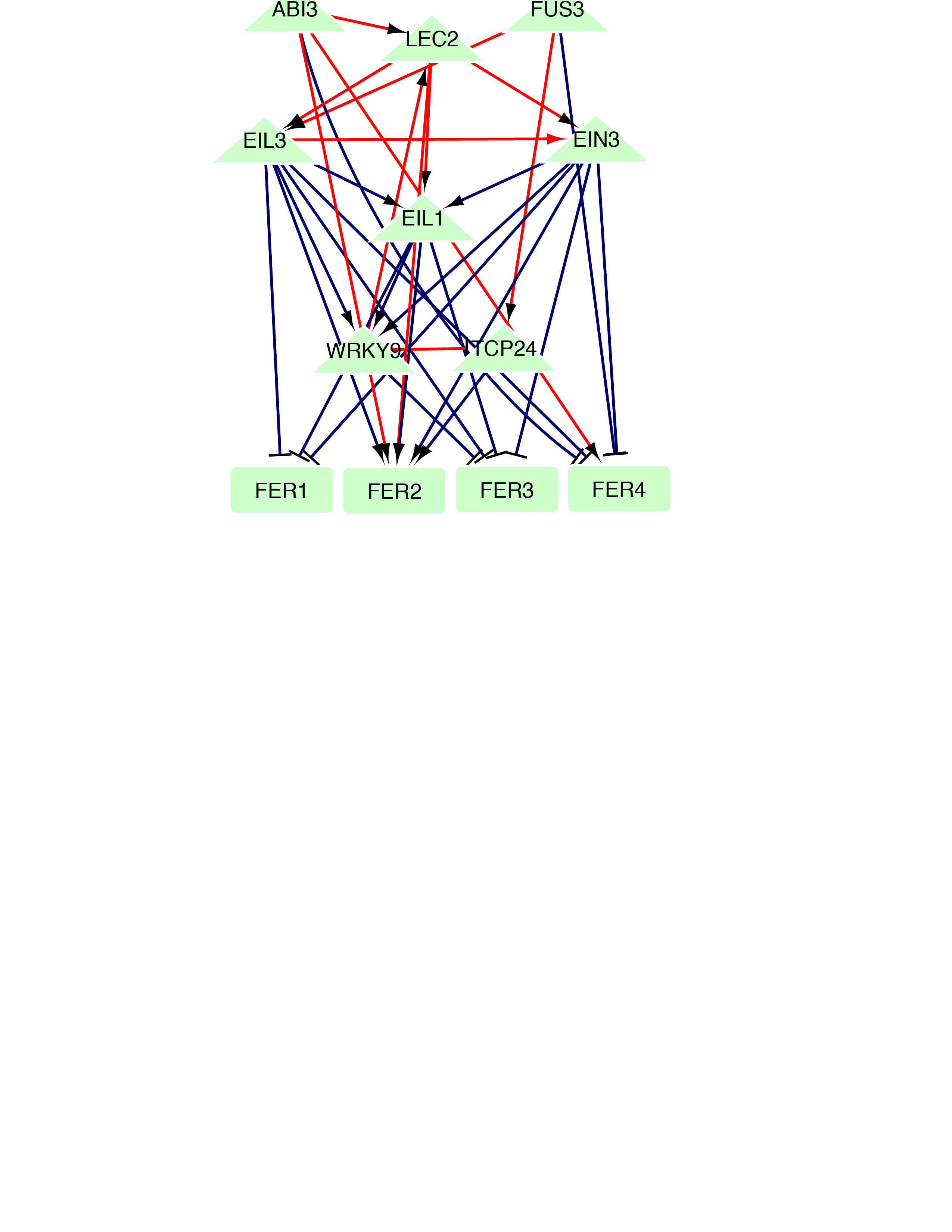

Supplement: Supplementary Figure 3 — Gene regulatory network controlling FERRITIN gene expression in seeds. We obtained a list of differentially expressed genes from seed transcriptome datasets of B3 mutants and searched for transcription factor-target gene interaction data in the plant cistrome database (O’Malley et al., 2016), the CIS-BP database (Weirauch et al., 2014) and the Arabidopsis Gene Regulatory Information Server AGRIS (Yilmaz et al., 2011) in order to build a regulatory network. Transcription factor-target gene pairs were further filtered by coexpression (expression correlation) during seed development (Schmid et al., 2005). From this network, we extracted regulatory interactions between FERRITIN genes and transcription factors and visualized the network in Cytoscape (Shannon et al., 2003). In the network, triangles represent transcription factors and rectangles represent genes. The edges connecting the nodes represent regulatory interactions from AGRIS (red edges) and CIS-BP (blue edges). Arrow edges represent positive regulation (activation) and T edges represent negative regulation (repression) based on expression correlation. [file Image_3.JPEG]

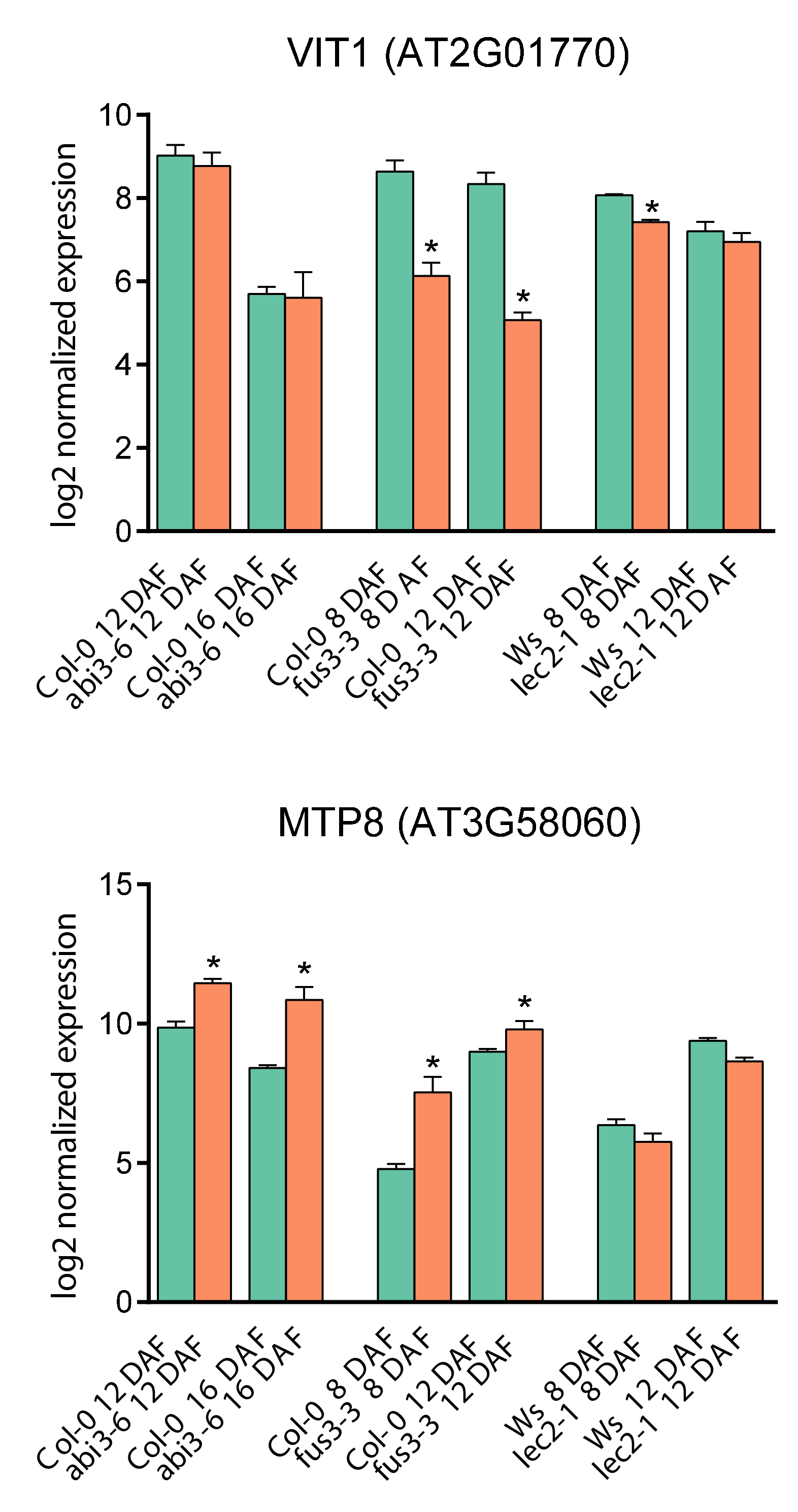

Supplement: Supplementary Figure 4 — Expression of VIT1 and MTP8 genes in B3 mutants. Transcriptome datasets from seeds of B3 mutants (Yamamoto et al., 2014) were obtained from the GEO database (GEO accession GSE61686). We show the log2 normalized expression for VIT1 and MTP8 in developing seeds dissected from siliques from the abi3-6, fus3-3, and lec2-1 mutants. DAF: days after flowering. The asterisks show means that significantly differ between mutants and their respective WT control (t-test, p ≤ 0.05). [file Image_4.JPEG]
