# Supplementary material for: B3 Transcription Factors Determine Iron Distribution and FERRITIN Gene Expression in Embryo but Do Not Control Total Seed Iron Content
Source: Front Plant Sci. 2022 May 6;13:870078. doi: 10.3389/fpls.2022.870078 (PMC9120844; doi:10.3389/fpls.2022.870078)
Supplement: Supplementary file 5 [file Table_1.docx]

| **Supplementary Table 1:**  **qRT-PCR primers** | | | |  |  |
| --- | --- | --- | --- | --- | --- |
| Name | Accession number | | Sequence 5’- 3’ | | Calibration Curve |
| qFER1 F | | AT5G01600 | TCCAACGATGGCCTCAAACG | | y = -3,4x + 14,818 |
| qFER1 R | |  | GAGAAACCGACGGAGAAGCA | |  |
| qFER2 F | | AT3G11050 | ATGTCGGCTTGAAAGGTTTCGC | | y= -3,587 + 14,304 |
| qFER2 R | |  | TCAAACTCAGAGACGGGCATCA | |  |
| qFER3 F | | AT3G56090 | AGCTGTAATCTCGCAGTTTGGT | | y= -3,446 + 16,492 |
| qFER3 R | |  | CCCAAGGAGCGTAATAGCTTGT | |  |
| qFER4 F | | AT2G40300 | GCATGGAGCTTGCTCTGTCACT | | y=-3,828x + 16,415 |
| qFER4 R | |  | TGATTGCTTCCACCTGCTCTGT | |  |
| qTIP41-like F | | AT4G34270 | TCATGCCAAGCTCATGGTTCCT | | y= -3,414x + 11,952 |
| qTIP41-like R | |  | TTGGTGCCTCATCTTCGCCAAA | |  |
